# Diclofenac Degradation by Immobilized *Chlamydomonas reinhardtii* and *Scenedesmus obliquus*

**DOI:** 10.1002/mbo3.70013

**Published:** 2024-12-17

**Authors:** Thamali Kariyawasam, Martin Petkovich, Bas Vriens

**Affiliations:** ^1^ Department of Geological Sciences and Engineering Queen's University Kingston Ontario Canada; ^2^ Beaty Water Research Center Queen's University Kingston Ontario Canada; ^3^ Department of Biomedical Engineering Queen's University Kingston Ontario Canada

**Keywords:** alginate beads, biodegradation, *Chlamydomonas reinhardtii*, diclofenac, *Scenedesmus obliquus*

## Abstract

Diclofenac (DCF), a commonly used anti‐inflammatory medication, presents environmental concerns due to its presence in water bodies, resistance to conventional wastewater treatment methods, and detection at increasing concentrations (ng/L to µg/L) that highlight DCF as a global emerging pollutant. While microalgae have been effective in degrading DCF in wastewater, immobilization into a matrix offers a promising approach to enhance treatment retention and efficiency. This study aimed to evaluate the efficacy of DCF removal using immobilized freshwater microalgae. Two algal species, *Chlamydomonas reinhardtii* (*Chlamydomonas*) and *Scenedesmus obliquus* (*Scenedesmus*), were tested for 6 days in both free and immobilized forms to determine if immobilized algae could degrade DCF comparably to free cells. The findings indicate that by Day 3, immobilized *Chlamydomonas* and *Scenedesmus* removed 78.0% and 80.1% of DCF, outperforming free‐cell cultures. Mixed cultures demonstrated synergistic effects, with removal amounts of 91.4% for free and 92.3% for immobilized systems. By Day 6, all conditions achieved complete DCF removal (100%). Mechanistic analysis showed 80.0% biodegradation and 20.0% bioaccumulation in free *Chlamydomonas* and 56.8% biodegradation with 43.2% bioaccumulation in *Scenedesmus*. Immobilization shifted pathways slightly: in *Chlamydomonas*, 61.6% of DCF removal occurred via biodegradation, 18.3% via bioaccumulation, and 20.1% via abiotic degradation. For *Scenedesmus*, immobilization achieved 45.6% biodegradation, 36.6% bioaccumulation, and 17.8% abiotic degradation, enhancing abiotic degradation while maintaining biodegradation efficiency. This research serves as a proof of concept for utilizing immobilized algae in DCF removal and suggests an avenue for improved wastewater treatment of emerging contaminants.

## Graphical abstract

Immobilized freshwater microalgae, specifically a combination of *Chlamydomonas reinhardtii* and *Scenedesmus obliquus*, exhibit superior efficiency in the removal of diclofenac from water compared to their free‐floating counterparts. This research validates the concept of employing immobilized algal cocktails to effectively degrade diclofenac in wastewater treatment processes. “Created with BioRender.com”.

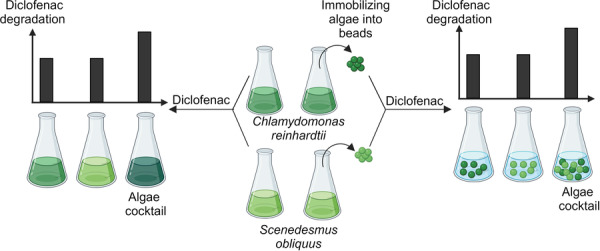

## 1 Introduction

DCF is a widely prescribed nonsteroidal anti‐inflammatory drug (NSAID) known for its efficacy in pain management and reduction of inflammation (Altman et al. 2015). However, its extensive and persistent presence in aquatic environments has raised significant environmental concerns (Sathishkumar et al. 2020). DCF is frequently detected in freshwater ecosystems worldwide, at concentrations ranging from nanograms to micrograms per liter (ng/L–µg/L) (Mirzaee et al. 2021). Despite its therapeutic benefits, DCF's resistance to conventional wastewater treatment processes, continual discharge from sewage effluents and inputs from agricultural runoff or pharmaceutical manufacturing facilities, contribute to its accumulation in surface waters (Vieno and Sillanpää 2014; Pan et al. 2024). Studies have shown adverse effects on aquatic organisms, including fish, mussels, and algae, even at low (ng/L) concentrations (Bouly et al. 2022; Harshkova et al. 2021). Furthermore, the long‐term ecological consequences of DCF contamination remain uncertain, warranting attention to mitigate potential environmental impacts in receiving aquatic ecosystems (Patel et al. 2019). Most conventional municipal water treatment methods cannot fully eliminate pharmaceuticals including DCF, highlighting the need for new and sustainable remediation approaches (Mussa et al. 2022; Hejna, Kapuścińska, and Aksmann 2022; Oluwole, Omotola, and Olatunji 2020).

Microalgae can fulfill a critical role in pharmaceutical contaminant removal from wastewater, owing to their capacity to uptake and metabolize a wide variety of organic pollutants such as antibiotics, hormones, and NSAIDs like diclofenac (Abdelfattah et al. 2022; Goh et al. 2022). However, in the realm of wastewater treatment, the effective utilization of microalgae presents a dual challenge: harnessing their potent bioremediation capabilities while ensuring their containment to prevent unintended environmental release. Additionally, microalgae require prolonged exposure to efficiently degrade DCF, as evidenced by varying removal efficiencies across different species, media, and incubation periods (summarized in Table 1). For instance, *Chlorella vulgaris* achieved 85.5% DCF removal in Bold Basal Medium (BBM) over 27 days (Sánchez‐Sandoval et al. 2022), while *Chlorella sorokiniana* reached 91.5% removal in BG‐11 media after 9 days at an initial concentration of 10 mg/L (Sharma et al. 2023). However, removal efficiency decreased with higher concentrations, such as 71.7% at 100 mg/L (Sharma et al. 2023). Similarly, anaerobically treated wastewater required 31 days for *Chlorella sorokiniana* to achieve 40%–60% removal of 147 µg/L DCF (de Wilt et al. 2016).

**Table 1:** Examples of diclofenac removed by algae species.

Culture condition	Initial diclofenac concentration	Microalgae	Cultivation system	Diclofenac removal efficiency (%)	Incubation time	References
BG‐11 media	10 mg/L	*Chlorella sorokiniana*	Batch cultures	91.5	9 days	Sharma et al. (2023)
	100 mg/L	*Chlorella sorokiniana*		71.7		
High Salt Medium	32.7 mg/L	*Chlamydomonas reinhardtii*	Batch cultures	37.7	4 days	Liakh et al. (2023)
Bold basal medium (BBM)	10 µg/mL	*Chlorella vulgaris*	Batch cultures	85.5	27 days	Sánchez‐Sandoval et al. (2022)
		*Nannochloropsis oculata*		80.5		
	8.7 µg/mL	*Scenedesmus acutus*		85.6		
		*Scenedesmus obliquus*		90.5		
BBM	25 mg/L	*Graesiella* sp.	Batch cultures	73	5 days	Ben Ouada et al. (2019)
	50 mg/L			42		
	75 mg/L			25		
Zarrouk medium	25 mg/L	*Picocystis* sp.		52		
	50 mg/L			28		
	75 mg/L			24		
Mann and Myers	25 mg/L	*Chlorella sorokiniana*	Bubbling column photobioreactors (PBRs) 300 mL capacity	30	10 days	Escapa et al. (2016)
		*Chlorella vulgaris*		21.6		
		*Scenedesmus obliquus*		79.1		
Anaerobically treated wastewater	147 ± 9 µg/L	*Chlorella sorokiniana*	Batch cultures	40–60	31 days	de Wilt et al. (2016)

Indigenous microalgae commonly found in wastewater treatment plants (WWTPs) include *Chlorococcum* sp., *Chlorella* sp., *Scenedesmus* sp., and *Tetradesmus* sp. (Pereira et al. 2018). Besides microalgae, bacterial communities are also present, predominantly from the phyla *Proteobacteria*, *Bacteroidetes*, *Acidobacteria*, *Firmicutes*, and *Nitrospirae*, with *Proteobacteria* typically being the most abundant (Numberger et al. 2019). Specific genera such as *Pseudomonas*, *Acinetobacter*, *Bacillus*, and *Nitrosomonas* that are present in WWTPs are known for their capabilities in breaking down complex pharmaceuticals including DCF (Xie et al. 2021). Whereas the pharmaceutical degradation efficiency varies significantly depending on the microbial species and environmental conditions, the degradation rates of DCF in conventional WWTPs are generally low (Vieno and Sillanpää 2014). Studies report removal amounts of DCF below 40% in standard treatment processes like activated sludge or anaerobic fermentation (Ma et al. 2020). Hence, this inefficiency leads to the presence of DCF in the effluents of WWTPs, subsequently affecting receiving surface waters.

Immobilization techniques have gained attention as a means to enhance the efficacy and longevity of microalgae‐based remediation systems (Ruiz‐Marin, Mendoza‐Espinosa, and Stephenson 2010). Immobilization of algae is a technique that restricts cell mobility by attaching the cells to a solid support or entrapping them within a polymer matrix (Girijan and Kumar 2019). Immobilization enables microalgae retention within a matrix, ensuring continuous contact with contaminants and enhancing removal efficiency (Melnikova et al. 2022; Encarnação et al. 2020; Mollamohammada, Aly Hassan, and Dahab 2021). In the treatment of wastewater from pharmaceutical manufacturing plants, immobilized algae have been shown to effectively remove antibiotics and other persistent organic pollutants (Obaid, Salman, and Kadhim 2023). For instance, immobilized *C. vulgaris* (*Chlorella*) demonstrated greater sulfamethoxazole tolerance and removal efficiency compared to suspended cells, promoting a symbiotic relationship with bacterial populations and enhancing contaminant degradation (Xie et al. 2020). Additionally, in aquaculture settings, immobilized algae systems are used to manage nutrient loads by removing excess nitrogen and phosphorus from water, thus maintaining water quality and preventing eutrophication (Obaid, Salman, and Kadhim 2023). For example, immobilized *Scenedesmus* was able to remove 90% of ammonium within 4 h and 100% of phosphate within 2 h from typical effluent, highlighting its potential for tertiary wastewater treatment (Chevalier and De la Noüe 1985). Moreover, Travieso et al. (1996) used immobilized *Chlorella* for secondary wastewater treatment in municipal treatment, demonstrating its efficacy over 6 months of operation. Furthermore, the utilization of microalgal cocktails, comprising multiple algal species, has demonstrated synergistic effects on pollutant removal, offering potential advantages over single‐species systems (Avila et al. 2021; Abdel‐Razek et al. 2019). These examples underscore the broad applicability and effectiveness of immobilized algae in various environmental and industrial contexts, making it a promising strategy for sustainable bioremediation and wastewater treatment.

Although the literature has explored the use of immobilized algae for bioremediation, a significant knowledge gap exists in the comparative analysis of free algae cells versus immobilized algae cells and also when using multiple algae in cocktails for bioremediation processes. While previous studies have investigated the free and immobilized approaches for bioremediation separately, there is a notable absence of direct comparisons between the two methods. This lack of comparative data and the algae cocktail approach limits our understanding of their relative performances and hinders the optimization of algal bioremediation techniques. A thorough comparative analysis of free algal cells, immobilized algal cells, and the algal combinatorial approach offers critical insights into their respective efficiencies, constraints, and potential applications across diverse remediation contexts. This comprehensive evaluation enhances our understanding of these methodologies, facilitating the optimization of algal‐based bioremediation strategies for various environmental scenarios.

In this study, we evaluate the efficiency of DCF removal by free and immobilized *Chlamydomonas* and *Scenedesmus*, two model microalgal species, when used individually and in combination. Through the immobilization of microalgae within a matrix and the utilization of a cocktail approach, we demonstrate their effective degradation of DCF, with the algae cocktail exhibiting a faster removal amount compared to free cells. Also, our findings demonstrate the potential of the algal cocktail remediation strategy for pharmaceutical contaminant removal.

## 2 Materials and Methods

### 2.1 Algal Cells and Culturing Conditions

The microalgae strains used in this study were *Chlamydomonas reinhardtii* (CC‐400) from Chlamydomonas Resource Center and *Scenedesmus obliquus* (UTEX 393) from UTEX Culture Collection of Algae. All the cultures were single colonies isolated before the experiments to have plated monocultures. Cells were maintained under a medium light level (50 μmol photons/m^2^/s) at 23°C on Tris‐acetate phosphate (TAP) medium (Harris 1989) containing 1.5% Bacto agar (purchased from Fisher Scientific, USA). DCF (purchased from Cayman Chemical Company Inc., USA) was dissolved in double distilled H_2_O (ddH_2_O) and added to cell cultures at the beginning of each experiment to obtain a final concentration of 150 µM (47.7 mg/L). For our experiments, we selected a DCF concentration of 150 µM (47.7 mg/L), which lies between the EC_10_ (32.7 mg/L) and EC_25_ (65.75 mg/L) values of DCF for *Chlamydomonas* as reported by Harshkova et al. (2021). This concentration was chosen after confirming that it did not impair cell growth compared to cells grown in TAP medium. Cell growth was assessed using hemocytometer‐based cell counts, which were conducted over a period of 6 days (Table A1). The results showed no significant difference in growth rates between the DCF‐treated cells and the TAP‐grown control cells for both *Chlamydomonas* and *Scenedesmus* cultures (Table A1).

### 2.2 Preparation of Liquid and Bead Cultures

**Figure 1:**
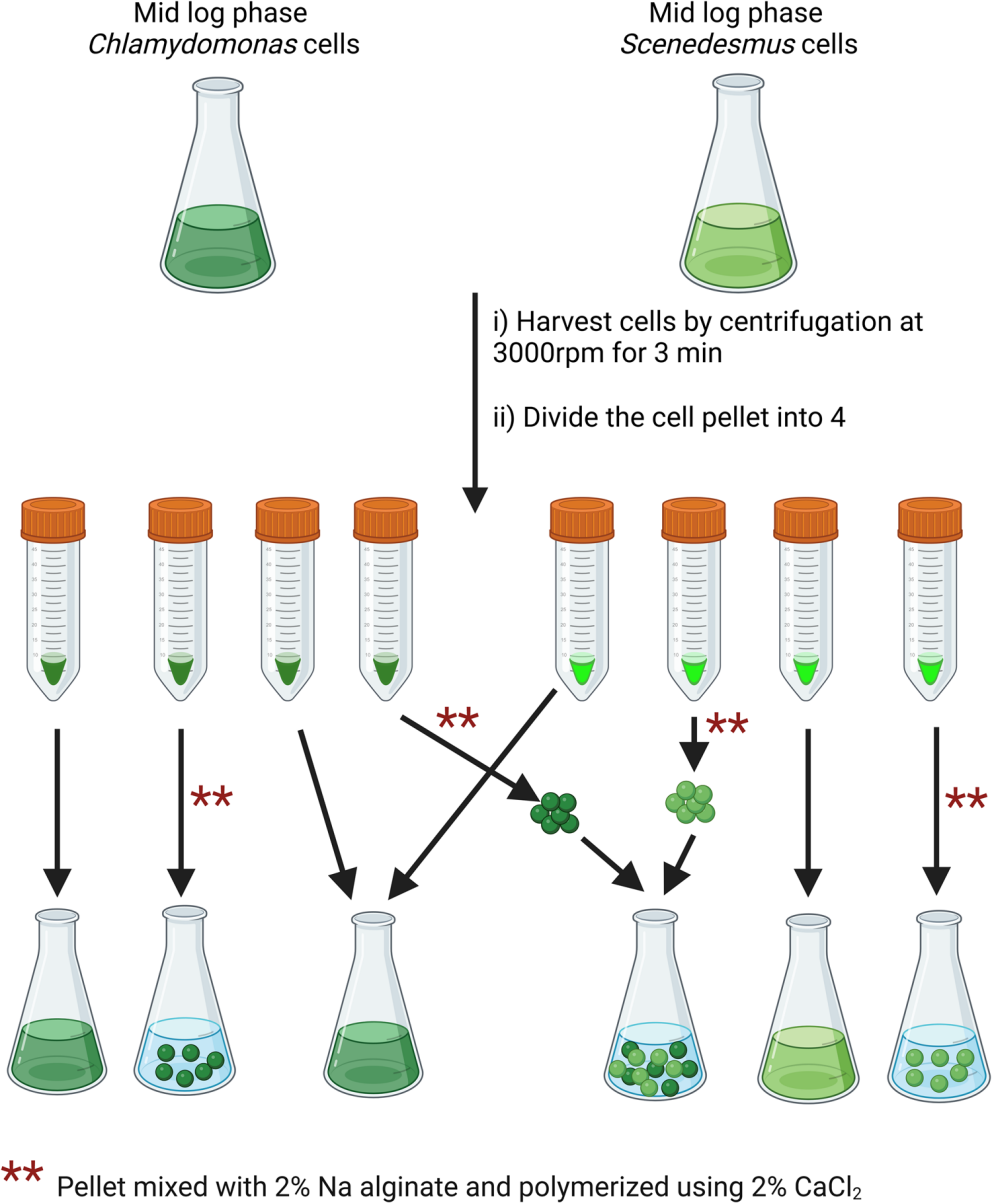
Schematic representation of the procedure used in the experimentation. *Chlamydomonas* and *Scenedesmus* were inoculated at 2 × 10⁵ cells/mL in 200 mL TAP media and grown under light with continuous shaking for 3 days until they reached mid‐log phase. Asynchronized cells were diluted to 4.5–4.7 × 10⁶ cells/mL and divided into four groups: two for liquid cultures and two for immobilization. For immobilization, pellets were resuspended in 2% Na‐alginate, and droplets were polymerized in 2% CaCl₂ to form ~4 mm beads (20 beads/culture). Beads were washed and introduced into DCF media. TAP media served as negative control, while TAP + 150 µM DCF without algae or with empty beads served as positive controls. “Created with BioRender.com.”

*Chlamydomonas* and *Scenedesmus* were inoculated in 2 × 10^5^ cells/mL concentration in 200 mL TAP cultures without agar and grown for 3 days under continuous light (50 μmol photons/m^2^/s) at continuous shaking (120 rpm). At the beginning of the experiment, the asynchronized cell population was diluted to a starting cell concentration between 4.5 × 10^6^ and 4.7 × 10^6^ cells/mL and divided into four sub‐populations (Figure 1). Two of the sub‐populations, were used in liquid/free culture experiments while the remaining two were used in immobilization. For immobilization, each pellet was resuspended in 5 mL of 2% (w/v) Na‐alginate (Sigma‐Aldrich, CA). The gel droplets were introduced gradually using a 1 mL micropipette into a 250 mL beaker with a 2% CaCl_2_ (≥ 97%, Sigma‐Aldrich, CA) solution, where they polymerized to form beads approximately 4 mm in diameter (Melnikova et al. 2022). Each culture had 20 beads. After polymerization, beads were washed three times with sterilized ddH_2_O water before introducing into the DCF media. TAP media without DCF and algae cells was used as the negative control, while TAP + 150 µM DCF without algae cells was used as a positive control. For the immobilization experiments, empty beads without algae in TAP + 150 µM DCF were used as a positive control. All the experiments were carried out under continuous light (50 μmol photons/m^2^/s) at continuous shaking (120 rpm). Data for each condition were collected from three biological replicates.

### 2.3 Measuring Algal Growth

For liquid cultures, 500 μL from each of the cultures were collected on Day 3 and Day 6 for cell counting and assessment of culture growth. For bead cultures, two beads per flask were collected and sacrificed and completely disintegrated in 10 mL of 4% NaHCO_3_ (Sigma‐Aldrich, CA) solution (w/v) (Mujtaba and Lee 2017) and the re‐suspended cells were used for counting. Cell counts for both liquid and disintegrated bead cultures were conducted using a hemocytometer chamber.

### 2.4 Measuring DCF Removal Efficiency

To measure DCF removal efficiency, 2 mL of each incubated culture was collected on Day 0, Day 3, and Day 6. All the samples were spun at 4200*g* for 5 min, and the supernatant was collected and filtered through 0.45 μm nitrocellulose filter before DCF measurements. Samples were analyzed by ISQ EM Single Quadrupole Mass Spectrometer instrument using positive ESI mode with an ion transfer tube temperature of 300°C and ion spray voltage of 3.0 kV. For DCF isolation, a C18 column (Hypersil ODS 50 × 4.6 mm 3‐Micron, Thermo Scientific) and mobile phase comprised of 90% acetonitrile and 10% water were used, run isocratically for 5 min with a flow rate of 1 mL/min at 40°C. The DCF peak area was used for calibration and % DCF removal efficiency in each sample was calculated using the following equation:

[(DCFd0−DCFdt)/DCFd0]×100,

where d0 and dt represent the DCF concentration at Day 0 and Day 3/Day 6, respectively.

### 2.5 DCF Migration and Distribution

The amount removed (*R*, %), migration, and distribution of DCF was calculated using the following equation:

$$R=B_{d}+B_{a}+B_{s}+\Delta R,$$

where *B*_d_, *B*_a_, and *B*_s_ represent the biodegradation, bioaccumulation, and biosorption rates (%) of DCF via microalgae, respectively, and Δ*R* denotes abiotic removal (%) of DCF. These parameters were calculated following the methodology outlined by Song et al. (2019). To determine *B*_d_, *B*_a_, and *B*_s_ on Day 6, the algae culture was centrifuged at 5000 rpm for 10 min and 1 mL of the supernatant was used to measure the residual amount of DCF. The resulting algae pellet was then shaken with 1 mL of methanol (5% v/v prepared in TAP, which is safe to use with cell wall‐less strains [Piasecki, Diller, and Brand 2009]) for 5 min, centrifuged again at 5000 rpm for 10 min, and the supernatant was collected to measure extracellular adsorption (*B*_s_). The remaining algae pellet was dissolved in a mixture of 1.5 mL dichloromethane and methanol (1:2 v/v), sonicated for 1 min, stored at −20°C overnight, and centrifuged at 5000 rpm for 10 min. The supernatant was collected to determine intracellular bioaccumulation content (*B*_a_). For the abiotically removed fraction (Δ*R*; primarily adsorption by alginate beads), the beads were removed from the solution, shaken in 2 mL of methanol, and centrifuged at 5000 rpm for 10 min. The supernatant was collected to measure the abiotic part (Δ*R*). The extraction of microalgae from the beads followed the procedure described for cell counting above (Mujtaba and Lee 2017).

### 2.6 Statistical Analysis

All assays were performed in at least three independent experiments. Data were expressed as mean ± SD. The GraphPad Prism 7 software (GraphPad Software, La Jolla, CA, USA) was used for statistical analysis. Two‐tailed Student's *t*‐tests were performed to evaluate the differences among groups. Differences among means were considered significant at *p* ≤ 0.05.

## 3 Results

### 3.1 Immobilization Does Not Impede Algae Growth

**Figure 2:**
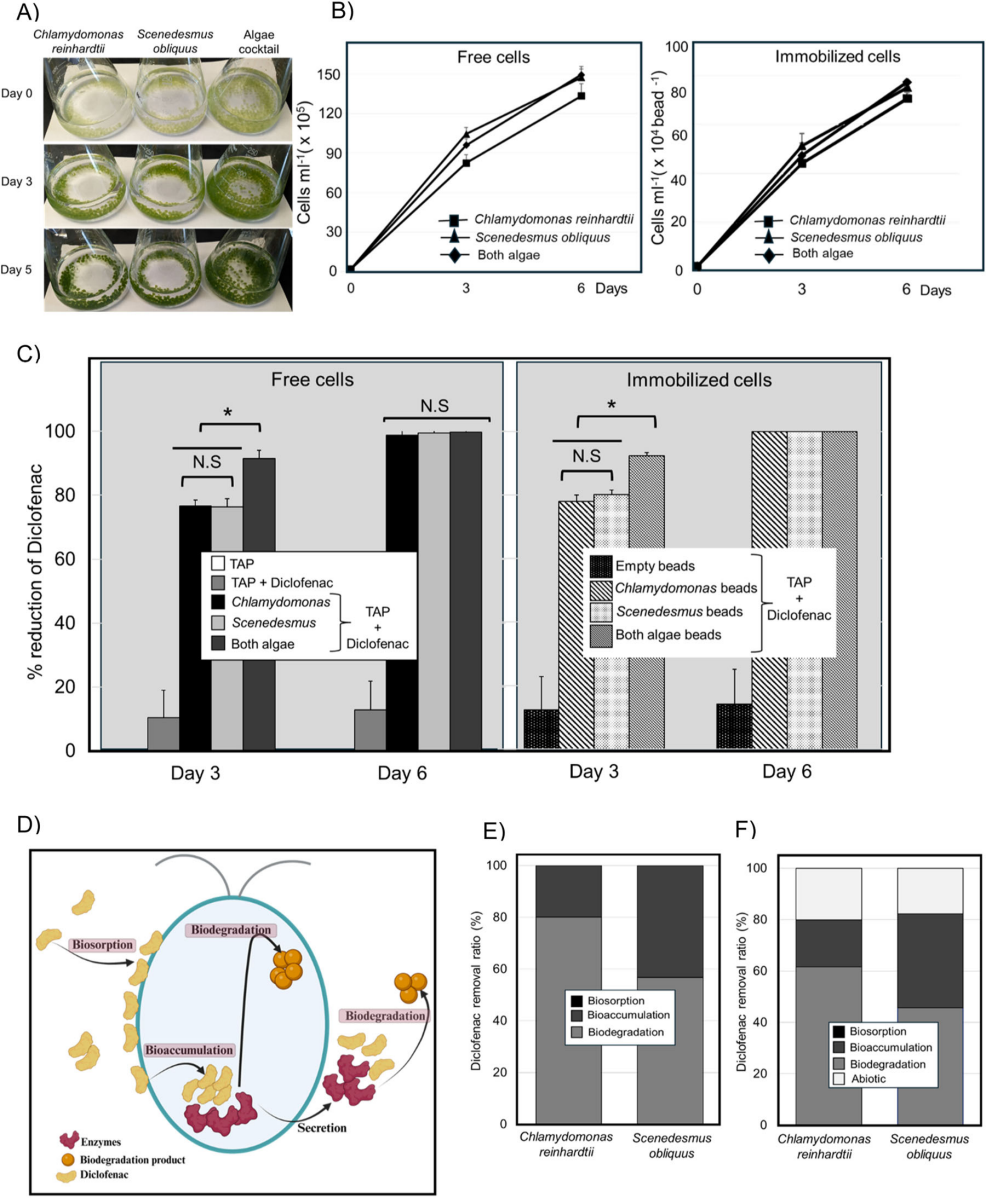
Growth and diclofenac removal in free and immobilized algae cultures. (A) alginate beads at the start and at the end of the experiment in *Chlamydomonas reinhardtii*, *Scenedesmus obliquus*, and algae cocktail (B) cell density of the free and immobilized cells at the start and end of the experiments. DCF removal efficiency in free and immobilized cells (C). By Day 3, Chlamydomonas and Scenedesmus immobilized counterparts achieved higher removal amounts than their free cells. Mixed cultures showed even higher efficiencies, compared to the monocultures. Complete removal of DCF was achieved in both free and immobilized cultures by Day 6. (D) An overview of three main mechanisms by which microalgae process organic pollutants. (E) Free and (F) immobilized Chlamydomonas and Scenedesmus removed DCF primarily via biodegradation with no biosorption in either culture. *p  ≤  0.05 in C. (D) “Created with BioRender.com”.

To assess whether immobilization affects algal growth, we compared the growth and survival rates of *Chlamydomonas* and *Scenedesmus* when immobilized in alginate beads versus their free‐floating counterparts in the presence of DCF (Figure 2A). We monitored cell density from Day 0 to Day 6 through cell counts. By Day 6, the total number of *Chlamydomonas* cells in liquid culture reached 1.33 × 10^8^ cells, whereas the immobilized algae achieved 1.55 × 10^8^ cells. Similarly, for *Scenedesmus*, the cell count in liquid cultures was 1.47 × 10^8^ cells, while immobilized cultures exhibited a slightly higher count of 1.50 × 10^8^ cells (Figure 2A,B). The slight variation in cell counts between immobilized and free‐floating cells was within the experimental margin of error (0.12 × 10^8^ to 0.08 × 10^8^ cells and *p* < 0.05), underscoring that immobilization in alginate beads maintains algal viability and proliferative capacity.

When examining mixed cultures, the total cell number in liquid cultures was 1.41 × 10^8^ cells, and in immobilized algal beads, it was 1.48 × 10^8^ cells. The combination of both algal species did not significantly enhance total cell numbers, suggesting potential competition for limited nutrients within the culture medium (Figure 2A,B). To substantiate this hypothesis, additional experimental data on nutrient uptake kinetics and medium composition over time would be valuable. Such information could confirm whether nutrient depletion occurs within the experimental timeframe, thereby explaining the observed cell number plateau in mixed cultures.

Overall, our observations indicate that the immobilization process does not impede algal growth. Both *Chlamydomonas* and *Scenedesmus* demonstrated growth rates comparable to their free‐floating counterparts, even under the stress of pharmaceutical contamination.

### 3.2 Immobilized *C. reinhardtii*, *S. obliquus*, and the Algae Cocktail Exhibit DCF Degradation Comparable to That of Free Cells

By Day 3, individual *Chlamydomonas* cultures in liquid media removed 76.6% of DCF, whereas immobilized *Chlamydomonas* achieved a slightly higher removal amount of 78.0%. Similarly, *Scenedesmus* in liquid culture removed 76.4% of DCF, while immobilized *Scenedesmus* demonstrated an improved removal efficiency of 80.1% (Figure 2C).

Despite the lack of a significant increase in cell numbers when combining the two algal species, the mixed algal cultures exhibited enhanced DCF degradation compared to individual cultures. The mixed liquid culture of *Chlamydomonas* and *Scenedesmus* achieved a DCF removal percentage of 91.4%, whereas the immobilized mixed culture demonstrated a slightly higher removal amount of 92.3%. These results suggest a synergistic effect in mixed cultures that enhances the degradation performance (Figure 2C).

By Day 6, we observed 100% removal of DCF in all tested cultures, including both free and immobilized, single and mixed algal cultures (Figure 2C). While prolonged incubation did not yield higher degradation rates, our data indicate that immobilized algae demonstrated slightly higher degradation rates during shorter incubation periods. This finding is particularly relevant for wastewater treatment applications, where the efficiency and speed of contaminant removal are crucial.

These findings indicate that immobilization does not compromise the algal ability to degrade DCF; in fact, it appears to enhance degradation efficiency, particularly in mixed cultures. The slight improvement in degradation rates for immobilized cells could be attributed to several factors, including more stable microenvironments within the alginate beads and potentially enhanced interactions between the algal cells and the contaminant discussed below under abiotic degradation.

### 3.3 DCF Migration and Distribution in Free and Immobilized Cells Are Comparable

Microalgae process organic pollutants through three main mechanisms: biodegradation, bioaccumulation, and biosorption (Dubey et al. 2023) (Figure 2D). To quantify DCF removal by biodegradation, biosorption, and bioaccumulation, we measured DCF concentrations on the outer surface and within microalgal cells at Day 6 samples (as described in Section 2.5). Given that there was 100% degradation of DCF by Day 6 in all samples (as determined by the residual DCF measurements), the amount removed (*R*) (equation from Section 2.5) was considered to be 100%. For free cell cultures, biodegradation was calculated by subtracting bioaccumulation and biosorption from *R* (Figure 2E). For immobilized samples, the abiotic removal was also subtracted from *R* when determining biodegradation **(**Figure 2F).

In free *Chlamydomonas* cultures, 80% (3.82 mg) of DCF was removed through biodegradation, while the remaining 20% (0.95 mg) was eliminated via bioaccumulation; notably, no significant contribution from biosorption was observed. Similarly, in free *Scenedesmus* cultures, approximately 56.8% (2.72 mg) of DCF underwent biodegradation, with 43.2% (2.06 mg) removed via bioaccumulation, and no discernible biosorption activity was calculated. Upon immobilization, the removal patterns shifted slightly: immobilized *Chlamydomonas* cells exhibited a distribution where 61.6% (2.94 mg) of DCF was degraded through biodegradation, 18.3% (0.87 mg) via bioaccumulation, and 20.1% (0.96 mg) via abiotic degradation. Conversely, in immobilized *Scenedesmus* cultures, biodegradation accounted for 45.6% (2.18 mg) of DCF removal, bioaccumulation for 36.6%, (1.74 mg), and abiotic degradation for 17.8% (0.85 mg). Notably, biosorption did not significantly contribute to DCF degradation in either free or immobilized algae cultures.

These findings underscore the varied mechanisms at play in DCF removal by algal species and highlight the potential efficacy of immobilization in altering removal pathways. The results (Figure 2E,F) underscore that biodegradation plays the key role in DCF removal in both free and immobilized groups which aligns with previous studies that have shown that biodegradation is the most effective way by which microalgae eliminate pharmaceuticals, including DCF (Norvill, Shilton, and Guieysse 2016; Cuellar‐Bermudez et al. 2017) and that immobilization does not hinder the biodegradation capabilities of microalgae. Instead, it may enhance degradation efficiency, particularly through abiotic removal effects facilitated by the alginate beads. The significant contribution of biodegradation and bioaccumulation to DCF removal highlights the metabolic versatility and potential of immobilized microalgae in wastewater treatment applications (further discussed in Section 4). Also, in combination with the negligible biosorption, appears to suggest that once internalized, DCF metabolism is quite fast. This means that the uptake of DCF, and not the intracellular metabolism, appears to be rate‐limiting to its overall degradation.

## 4 Discussion and Conclusions

While immobilized algae have been studied for bioremediation, there is a lack of direct comparisons with free algae cells and the use of algae cocktails, which limits the optimization of algal bioremediation techniques. A comparative analysis of free and immobilized algae, as well as algae combinations, will provide essential insights into their efficiencies and applications, improving algal‐based bioremediation strategies for various environmental contexts. Our study aimed to address this knowledge gap by investigating the efficacy of immobilized microalgae compared to their free‐floating counterparts individually and when combined. Specifically, we focused on *C. reinhardtii* and *S. obliquus*, two widely studied algal species known for their bioremediation potential and widespread use in wastewater treatment technologies (El‐Sheekh et al. 2023).

Our results show that immobilized algae exhibit comparable efficacy to free cells in removing pharmaceutical contaminants from wastewater. This is particularly noteworthy as it suggests that immobilization does not compromise the biodegradation capabilities of microalgae. Furthermore, we observed a synergistic effect when combining *Chlamydomonas* and *Scenedesmus*, resulting in enhanced removal efficiency compared to using each species individually. This highlights the potential for leveraging the inherently present microbiome diversity within immobilized systems to maximize remediation performance.

While previous research has explored the benefits of immobilization primarily in the context of nutrient absorption (Melnikova et al. 2022), our study contributes by focusing on pharmaceutical contaminant degradation. This expands the scope of understanding regarding the applicability of immobilized algae in wastewater treatment scenarios. We encapsulated the algae in alginate beads, a method that offers several advantages. Immobilized cells occupy less space, are easier to handle, and can achieve higher cell densities, allowing for repetitive use in product creation, and enhancing both the adsorption capacity and bioavailability of algal biomass (Carbone et al. 2020; Eroglu, Smith, and Raston 2015). Furthermore, immobilization has been shown to strengthen operational stability by preventing cell drift, increasing reaction rates due to higher cell densities, and facilitating growth and easy harvesting (Obaid, Salman, and Kadhim 2023). Additionally, immobilization offers protection against harsh environmental conditions such as metal toxicity, high salinity, pH fluctuations, and product inhibition (Han et al. 2022). This technique also safeguards aging cultures from photoinhibition, allows for increased biomass concentrations, and ensures less destructive cell recovery. Immobilized systems also protect microalgae from external threats, including predators and growth inhibitors (Nair, Senthilnathan, and Nagendra 2019; Lee et al. 2020). These benefits collectively underscore the potential of immobilization for enhancing the efficacy and sustainability of bioremediation processes.

Based on the previous study by Song et al. (2019) on the removal of Florfenicol (FF), a widely used veterinary antibiotic, by the microalgae, *Chlorella* sp. found that at a concentration of 46 mg/L, biodegradation in *Chlorella* sp. was the sole removal pathway, achieving 97% efficiency. However, as the concentration increased, FF began to show bioaccumulation and biosorption. At 159 mg/L, the total removal decreased to 74.7%, with biodegradation, bioaccumulation, and biosorption contributing 72.0%, 1.3%, and 1.4%, respectively. In our study, the efficiencies and fractions of DCF removal through biodegradation, bioaccumulation, biosorption, and abiotic removal were determined when cells were exposed to a DCF concentration of 150 µM. Nonetheless, variations in DCF concentrations can influence the metabolic activity of the microalgae, similar to the observations by Song et al. (2019), potentially altering the balance between biodegradation, bioaccumulation, and biosorption processes, which warrants further study. At lower DCF concentrations, bioaccumulation efficiency may increase as cells have a higher capacity to absorb and store the contaminant (Liakh et al. 2023). Conversely, at higher concentrations, the cells might experience toxicity, reducing their overall metabolic activity and thus decreasing biodegradation efficiency (Ben Ouada et al. 2019). Additionally, the abiotic removal might also vary with different DCF concentrations, as higher contaminant levels could enhance the adsorption capacity of the immobilization matrix, leading to greater abiotic degradation. Understanding these dynamics is crucial for optimizing bioremediation strategies in wastewater treatment. Tailoring the exposure concentrations of contaminants can potentially maximize the removal efficiencies of specific pathways (biodegradation, bioaccumulation, biosorption, and abiotic removal). Further research should systematically study the effects of various DCF concentrations on removal efficiencies to establish robust models for predicting microalgae performance in different environmental conditions. Hence, this approach will enhance the practical applicability of microalgae‐based treatment systems in diverse wastewater scenarios.

In conclusion, our study provides compelling evidence for the viability of immobilized algal systems as a means of efficiently removing pharmaceutical contaminants from wastewater. The observed synergistic effects and comparable performance between immobilized and free cells emphasize the potential of this approach for achieving more sustainable and effective bioremediation solutions. Moving forward, future research endeavors could delve deeper into elucidating the underlying mechanisms driving enhanced degradation efficiency and optimizing conditions for broader‐scale implementation in real‐world wastewater treatment. Overall, our study contributes to the advancement of sustainable wastewater treatment technologies and addresses environmental challenges associated with pharmaceutical contamination in aquatic ecosystems.

## Author Contributions

**Thamali Kariyawasam:** conceptualization, investigation, writing–original draft, methodology, validation. **Martin Petkovich:** writing–review and editing, funding acquisition, supervision. **Bas Vriens:** funding acquisition, writing–review and editing, project administration, supervision.

## Ethics Statement

The authors have nothing to report.

## Conflicts of Interest

The authors declare no conflicts of interest.

## Supporting information

Supporting information.

## Data Availability

The data that supports the findings of this study are available in the supplementary material of this article.

## 1

**Table A1:** *Chlamydomonas* and *Scenedesmus* cell growth in TAP and TAP + 150 µM DCF.

Media	Cell line	Cell counts (**×** 10^5^ cells/mL)			
		Day 0	Day 2	Day 4	Day 6
TAP	*Chlamydomonas*	23.3 ± 2.9	38. 3 ± 7.6	83.3 ± 7.6	131. 7 ± 10.4
	*Scenedesmus*	21.67 ± 1.5	39.3 ± 2.1	85 ± 2.6	130.3 ± 2.5
TAP + DCF	*Chlamydomonas*	26. 3 ± 1.5	39 ± 3.6	86. 7 ± 1.5	133. 3 ± 7.6
	*Scenedesmus*	21.33 ± 3.1	36.3 ± 3.8	85.3 ± 2.5	120.3 ± 1.5
